# Reduced levels of dopamine and altered metabolism in brains of HPRT knock-out rats: a new rodent model of Lesch-Nyhan Disease

**DOI:** 10.1038/srep25592

**Published:** 2016-05-17

**Authors:** Stephen Meek, Alison J. Thomson, Linda Sutherland, Matthew G. F. Sharp, Julie Thomson, Valerie Bishop, Simone L. Meddle, Yoann Gloaguen, Stefan Weidt, Karamjit Singh-Dolt, Mia Buehr, Helen K. Brown, Andrew C. Gill, Tom Burdon

**Affiliations:** 1 The Roslin Institute and R(D)VS, University of Edinburgh, Easter Bush, Midlothian, EH25 9RG, Scotland; 2 Central Bioresearch Services, University of Edinburgh, Chancellor’s Building, 49 Little France Crescent, Edinburgh EH16 4SB, Scotland; 3 Glasgow Polyomics, College of Medical, Veterinary and Life Sciences, University of Glasgow, Wolfson Wohl Cancer Research Centre, Garscube Campus, Bearsden, G61 1QH, Scotland

## Abstract

Lesch-Nyhan disease (LND) is a severe neurological disorder caused by loss-of-function mutations in the gene encoding hypoxanthine phosphoribosyltransferase (HPRT), an enzyme required for efficient recycling of purine nucleotides. Although this biochemical defect reconfigures purine metabolism and leads to elevated levels of the breakdown product urea, it remains unclear exactly how loss of HPRT activity disrupts brain function. As the rat is the preferred rodent experimental model for studying neurobiology and diseases of the brain, we used genetically-modified embryonic stem cells to generate an HPRT knock-out rat. Male HPRT-deficient rats were viable, fertile and displayed normal caged behaviour. However, metabolomic analysis revealed changes in brain biochemistry consistent with disruption of purine recycling and nucleotide metabolism. Broader changes in brain biochemistry were also indicated by increased levels of the core metabolite citrate and reduced levels of lipids and fatty acids. Targeted MS/MS analysis identified reduced levels of dopamine in the brains of HPRT-deficient animals, consistent with deficits noted previously in human LND patients and HPRT knock-out mice. The HPRT-deficient rat therefore provides a new experimental platform for future investigation of how HPRT activity and disruption of purine metabolism affects neural function and behaviour.

Lesch-Nyhan Disease (LND) is a neurological disorder in humans that primarily affects men and is caused by mutations in the X-linked *Hprt* gene encoding hypoxanthine phosphoribosyltransferase (HPRT)^1^,^2^. This protein is a key component of the purine salvage pathway and its loss in LND patients is associated with excessive uric acid (hyperuricaemia), which causes kidney damage and gout in affected patients. LND is also associated with deficits in motor and cognitive capabilities and self-harming behaviour. Many symptoms can be controlled with drugs that reduce the level of uric acid in the blood (allopurinol). However, this treatment does not satisfactorily alleviate neurological or behavioural problems, implying that chronic hyperuricaemia is not directly responsible for these symptoms^3^. Moreover, a subset of patients carrying mutations in *HPRT* suffers from hyperuricaemia but does not exhibit significant neurological deficits. This could imply that loss of HPRT activity affects other important molecular pathways, a notion that is supported by the finding that the basal ganglia within the brains of LND patients have markedly reduced levels of the neurotransmitter dopamine^4^. The significance of this deficit is not entirely clear, but depletion of catecholamine- (including dopamine) containing neurons in the brains of newborn rats can also induce self-injurious behaviour upon administration of L-DOPA^5^. Although treatment with dopamine receptor antagonists lessened symptoms in these animals, treatment of LND patients with receptor antagonists had no beneficial effects on self-injurious behaviour. There is, therefore, a pressing need to develop new animal models to investigate the biochemistry and biology associated with loss of HPRT.

The location of *Hprt* on the X-chromosome, and the ability to select for loss or gain of HPRT function through selection using synthetic nucleotide analogues, made the *Hprt* gene an attractive target for the first gene knock-out experiments in mice using embryonic stem cells^6^,^7^. The resulting HPRT knock-out mice had reduced levels of dopamine in the striatum, consistent with data obtained from human patients^4^,^8^,^9^,^10^. However, loss of HPRT activity in mice did not induce the classic neurological symptoms associated with LND. This has been attributed to a number of factors, including species-specific differences in the operation of the purine salvage pathway that may limit neurological damage in the mouse brain^11^. It is also possible that subtle disturbances of neuronal development and function might be more readily detected in a behaviourally sophisticated experimental animal like the rat.

The laboratory rat is a popular experimental animal in many biological studies due to its convenient size, physiology and genetics, and is widely regarded as a useful model for brain and behavioural studies^12^,^13^. Although targeted genetic modification in rats to study gene function and model human disease has lagged behind that performed in the mouse, recent innovations using embryonic stem cells and gene-specific synthetic endonucleases have reignited interest in applying targeted genetic engineering in the rat^14^,^15^,^16^,^17^. In this report we describe the generation of a line of HPRT-deficient rats that exhibit metabolic and dopamine deficits associated with LND and should, therefore, provide a useful alternative experimental foundation for pharmacological and genetic studies into how HPRT deficiency affects the nervous system and behaviour.

## Results

### Generation of HPRT-deficient transgenic rats

**Figure 1:**
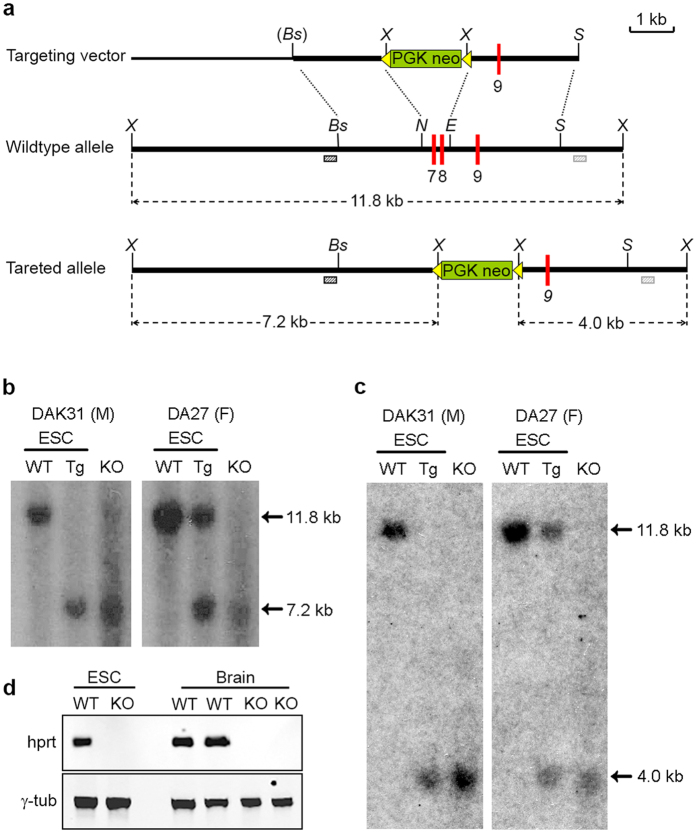
Generation of HPRT-deficient rats. (**a**) Structure of the HPRT targeting vector (top), the wild-type *Hprt* allele (middle) and targeted allele (bottom), resulting from replacement recombination at the dotted lines. The null allele was created by substitution of exons 7 and 8 with an *frt*-flanked PGKneo selection cassette (green box with yellow arrows). Exons are depicted by red boxes, non-exon–containing chromosomal, and cloned genomic DNA sequence is shown by a thick black line and pBluescript plasmid sequence by a thin black line. Restriction enzyme sites BstBI (*Bs*), *Eco*RV (*E*), *Nde*I (*N*), *Sac*I (*S*) and *Xba*I (*X*) are indicated. 5′ and 3′ probe sequences (dark and light hashed boxes respectively), external to the homology arms were used for Southern blot screening. The size of expected products are shown by dotted arrows. (**b**) Southern blot analysis using the 5′ external probe on genomic DNA of wildtype (WT) ES cells, targeted (Tg) ESC sub-clones and HPRT KO rats from 2i + LIF-derived DAK31 and 1i-derived DA27 cell lines (left and right panel respectively). (**c**) Southern blot analysis using the 3′ external probe on genomic DNA samples as described for (**b**). (**d**) Western blot analysis of HPRT protein expression in male wild-type and HPRT knock-out (KO) ESC lines, and brains of two wild-type rats (SD and DA) and two HPRT KO male rats.

Guided by previous reports, we designed a DNA targeting construct that inactivates HPRT function through deletion of exons 7 and 8 of rat *Hprt*^18^,^19^. The *Hprt* targeting vector contained 2.5 kb 5′ and 2.2 kb of 3′ genomic DNA surrounding a phosphoglycerate kinase (PGK) promoter-driven neomycin selection cassette flanked by frt recombination sites, replacing exons 7 and 8 of rat *Hprt* (Fig. 1a). We electroporated two germline competent DA (dark agouti) rat ESC lines with the linearised targeting vector: DAK31 is a male line derived in 2i + LIF culture^20^ and DA27 is female line derived in our laboratory using MEK inhibitor only (1i) culture medium^21^. Two days after electroporation ESCs were switched to medium containing the aminoglycoside G418, and after a further 7–9 days of selection, stably-transfected G418-resistant ESC colonies were picked and expanded. The clones generated from the male DAK31 cell line were plated in duplicate and one set was treated with 6-thioguanine (6-TG). This purine analogue is converted by HPRT to 6-thioguanine monophosphate which inhibits the synthesis and utilisation of purines and cell growth, thereby allowing the functional identification of HPRT-deficient clones. PCR genotyping and subsequent southern blot analysis, using 5′ and 3′ external probes from genomic regions flanking the 5′ and 3′ homology arms, identified one DAK31 and four DA27 clones carrying a correctly disrupted *Hprt* gene (*Hprt*^Δex7,8^). This represented targeting frequencies (number of targeted colonies/cells electroporated) of 0.1 × 10^−6^ and 0.4 × 10^−6^ for DAK31, and DA27 lines respectively, which are comparable with those previously obtained with Fischer F344 and SD rat ES cell lines^19^.

Based on ESC colony morphology, embryoid body differentiation^22^, and retention of a karyotype similar to that of the parental cell line, we selected targeted clones DA27-E1 and DAK31-4B12 for blastocyst injection to generate ESC-derived chimaeras. The DA27-E1 and DAK31-4B12 clones produced coat colour chimaeras at a frequency of 48% and 26% of live born pups generated from embryo transfers, respectively (Supplementary Table S1). The male DAK31-4B12 chimaeras were mated with SD females and resulting F1 germline females were bred with SD males to transmit the *Hprt*^Δex7,8^ allele to male HPRT-deficient rats. (Fig. 1b,c). Male pups from a female DA27-E1 chimaera carrying the ESC *Hprt*^Δex7,8^ allele were also identified (Fig. 1b,c). Western blots of rat brain tissue probed with anti HPRT antibody confirmed the absence of HPRT protein in male rats carrying the targeted *Hprt*^Δex7,8^ allele (Fig. 1d). HPRT-deficient male rats from both clones appeared healthy and were fertile. DAK31-4B12 derived animals were used to establish a line of *Hprt*^Δex7,8^ transgenic rats. These rats attained the same weights as matched wild-type littermates (Supplementary Fig. S1) and displayed normal caged behaviours.

### Metabolomic analysis of the brains of HPRT-deficient rats

**Figure 2:**
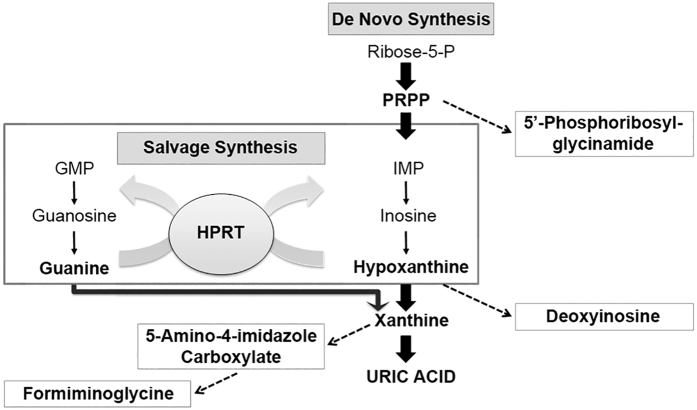
The role of HPRT in the purine salvage pathway. HPRT plays a central role in generating purine nucleotides by catalysing the conversion of hypoxanthine and guanine to inosine monophosphate (IMP) and guanine monophosphate (GMP) respectively. The *de novo* purine synthesis pathway via phosphoribosylpyrophosphate (PRPP) is also highlighted.

**Figure 3:**
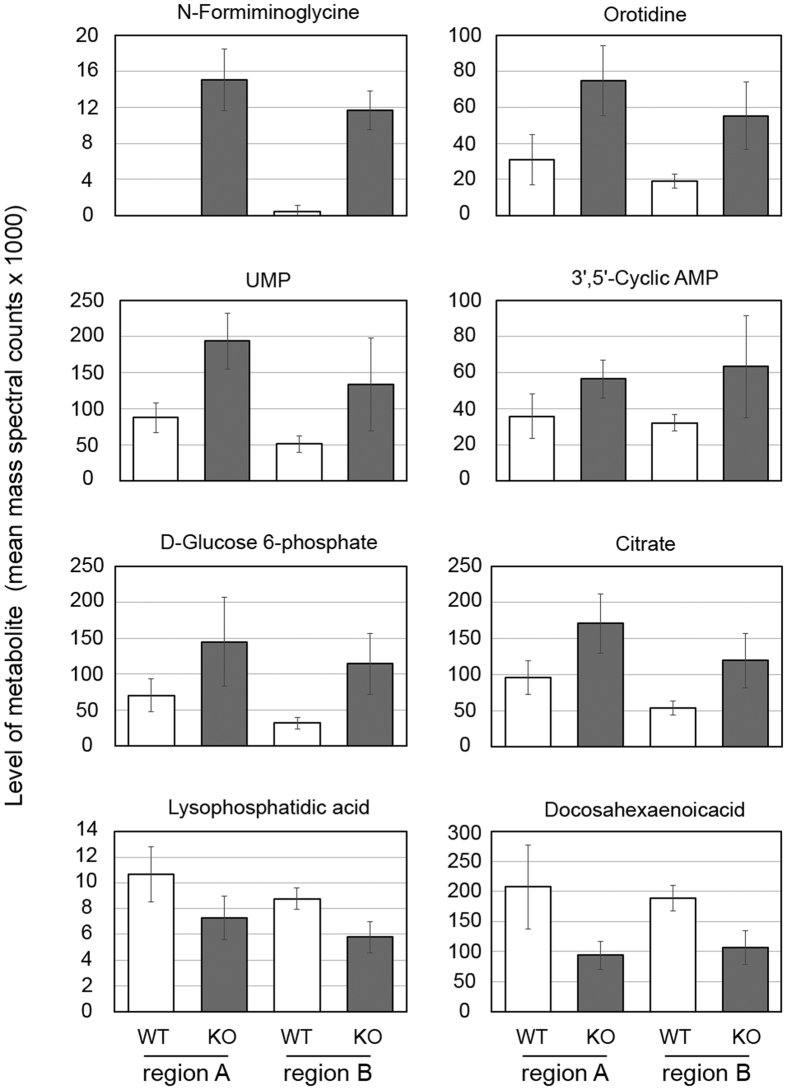
Altered brain metabolism in brains of HPRT-deficient rats. Graphs describing LC-MS analysis of select metabolites that differ between wild-type and HPRT-deficient rat brains in either region A (containing hippocampus, overlying cortex, striatum and hypothalamus) and/or region B (containing olfactory bulb and pre-frontal cortex). The graphs represent mean mass spectral counts +/− SD obtained from analysis of brains from 4 HPRT wild-type and 4 HPRT KO male rats. The values of most metabolites in WT and KO animals for a particular region differed (p < 0.05). Exceptions were UMP and 3′,5′-Cyclic AMP in region B, and D-Glucose 6-phosphate in region A.

Loss of HPRT has direct effects on purine synthesis, principally through the loss of the salvage pathway that converts hypoxanthine and guanine to inosine monophosphate and guanine monophosphate, respectively (Fig. 2). In Lesch-Nyhan patients lacking HPRT activity, the resulting excess hypoxanthine and xanthine leads to elevated levels of uric acid^1^,^2^. By contrast, in most other animals including rats, the presence of an active uricase enzyme limits the build-up of uric acid^23^. Indeed, the levels of uric acid we measured in HPRT KO rats were indistinguishable from wild-type animals and similar to basal levels reported previously^24^ (Supplementary Fig. S2). The loss of the purine salvage pathway, however, leads to increased levels of *de novo* purine synthesis and is expected to have wider consequences for other metabolic processes. To obtain an overview of how HPRT loss affects metabolism in the rat brain we compared the metabolic profile of two regions of wild-type and HPRT knock-out (KO) male rat brains (Supplementary Fig. S3) by liquid chromatography-mass spectrometry (LC-MS). The central area (region A) contained the hippocampus, overlying cortex, striatum and hypothalamus, and encompassed areas of the brain that in HPRT-deficient mice have previously been shown to have reduced levels of dopamine^8^,^10^,^25^. The forebrain area largely contained olfactory bulb and cortex (region B). Brain samples from four wild-type and four HPRT KO male rats were homogenised in chloroform/methanol solution and snap frozen in liquid nitrogen for storage before LC-MS analysis. The metabolic profiles of brain extracts from wild-type and KO rats unambiguously identified 69 core metabolites and assigned putative identities to an additional 675 compounds based on accurate mass measurements and retention time predictions, from which elemental compositions were calculated^26^ (Supplementary Table S2). To identify metabolites that were affected consistently by the loss of HPRT activity, we compared the areas under the peaks in LC-MS extracted ion chromatograms of the putative metabolites. The levels of 36 metabolites were altered in both regions A and B within HPRT-deficient brains (Supplementary Table S3). Two compounds, citrate and orotidine, were positively assigned through matches with standards whilst the others were predicted from accurate mass/retention time data. To confirm correct assignment of identities to the other 34 compounds, we reanalysed samples by targeted MS/MS analyses, searched the resulting data against publically-available spectral libraries and also submitted data to the *in silico* calculation tool MetFrag^27^. Twenty metabolites had elements within their MS/MS spectra that were consistent with molecular assignments made in the initial LC-MS analysis (Table 1). Within this set the level of N-formiminoglycine, a derivative of the HPRT substrate hypoxanthine, increased in HPRT-deficient rat brains (Fig. 2). Deoxyinosine, 5-amino-4-imidazole carboxylate and 5′-phosphoribosyl-glycinamide, which are also associated with purine metabolism, were identified in the original LC-MS analysis as consistently higher in HPRT KO rat brains, but MS/MS spectra for these compounds could not be acquired. Interestingly, compounds exhibiting statistically significant increases in one or other of the brain regions of HPRT KO animals included the pyrimidine derivatives UMP and CMP, the second messenger cAMP and glucose-6-phosphate (Fig. 3 and Supplementary Tables S3–5). These increases, and those of citrate and orotidine, were accompanied by reduced levels of lipids and fatty acids in HPRT KO rats (Fig. 3 and Supplementary Tables S3–5). Therefore, the loss of HPRT activity in rats leads to altered levels of compounds associated with nucleotide metabolism and wider changes in general metabolism in the brain.

### Analysis of the level of monoamines in brains of HPRT-deficient rats

**Figure 4:**
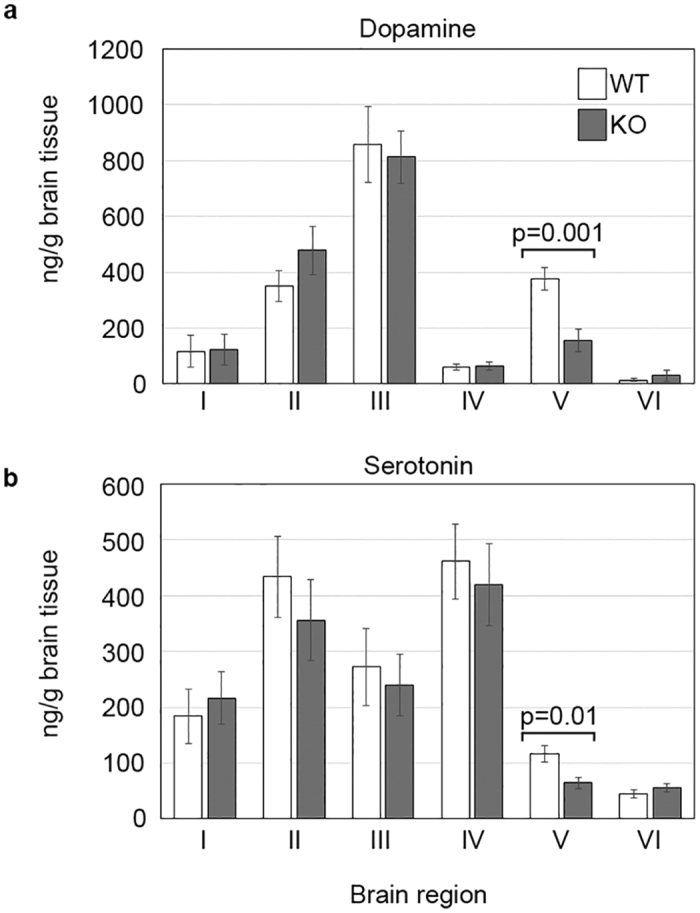
Effect of HPRT deficiency on the levels of dopamine and serotonin in rat brain. HPLC analysis of **(a)** dopamine and **(b)** serotonin in regions I–VI in the brains of wild-type and HPRT knock-out (KO) rats. (I: Olfactory bulb; II: hypothalamus; III: Pre-frontal Cortex; IV: Midbrain; V: Hippocampus, cortex and striatum; VI: Cerebellum. Y axis values represent means obtained from analysing 9 HPRT wild-type and 7 HPRT KO brain samples +/− SD. White and grey bars represent wild-type control and KO values, respectively.

LND and HPRT deficiency in mice is associated with loss of dopamine in areas of the brain^4^,^8^,^9^,^10^. To examine how loss of HPRT activity in the rat also affects the level of dopamine and its metabolites, or other monoamine neurotransmitters, we performed targeted HPLC-MS analysis on acetonitrile extracts prepared from 6 regions (I–VI) within the brains of nine wild-type and seven HPRT KO deficient males rats (Supplementary Fig. S4). Variation in the levels of the neurotransmitters tested (dopamine, dihydroxyphenylacetic acid, epinephrine, norepinephrine, tryptophan, serotonin, kynurenine, hydroxykynurenine, hyrdoxyanthranilic acid, and γ-aminobutyric acid) were observed between the different brain regions, but in most cases these did not differ significantly between wild-type and KO rats (Supplementary Fig. S5, Table S6). In contrast, dopamine and serotonin levels in region V containing the hippocampus and striatum were reduced by as much as 50% in comparison to control brain samples (Fig. 4). To determine whether the loss of dopamine was associated with changes in dopaminergic neurons or reduced levels of tyrosine hydroxylase (TH), a key enzyme in the synthesis of dopamine, we performed TH immunostaining on coronal sections of male wild-type and knock-out rat brains (Supplementary Fig. S6). The intensity and distribution of TH staining in KO rat brain sections was normal and similar to the wild-type controls, indicating that loss of dopamine was unlikely to be attributable to reduced levels of TH or TH-expressing neurons, and is in line with similar results previously reported for HPRT-deficient mice^10^,^25^.

## Discussion

Mutation of the *HPRT* gene causes severe neurological deficits in Lesch Nyan patients, and recapitulating this genetic lesion in mice has provided insights into some aspects of this debilitating disease^23^. Mutation of the *Hprt* gene has also served as a useful component in genetic engineering protocols because of the gene’s accessibility to transgene integration by homologous recombination, amenability to positive and negative selection schemes, and neutral character as a “harbour” for regulated transgene expression^28^,^29^. Here we have described targeted disruption of *Hprt* in independent rat ESC lines propagated in either the standard dual inhibitor (2i + LIF) or a single inhibitor (1i)-type culture system, and used the targeted clones to generate transgenic rats. We have established that brains of HPRT-deficient rats exhibit changes in levels of neurotransmitters and metabolites, and therefore propose that HPRT knock-out rats represent a new experimental platform to investigate the function of HPRT in the brain and LND.

The first successful derivation of rat ESC cultures was built on the discovery that dual chemical inhibition of MEK and GSK3 signalling pathways blocks mouse ESC differentiation^15^,^16^,^30^. This two-inhibitor (2i) culture system facilitated efficient establishment of rat ESC lines and allowed the implementation of gene targeting in the rat by homologous recombination^17^,^19^,^31^. Compared with mouse stem cell lines, however, rat ESC cultures were less stable, partly due to their different pattern of response to GSK3 inhibition in 2i-type medium^21^,^32^. Indeed, we previously showed that efficient *de novo* derivation and expansion of rat ESC lines could be achieved using the MEK inhibitor alone (1i culture) on fibroblast feeder cells, obviating the requirement for chemical inhibition of GSK3^21^. We have extended this observation by showing that a cell line propagated in this 1i condition can be genetically modified, undergo clonal selection/expansion and still retain the capacity to transmit a targeted mutation through the rat germ line. This highlights the scope for tuning the standard 2i culture regimen to assist the derivation of rat ESC from more refractory rat strains, or provide opportunities for differential priming of ESC signalling prior to exit from pluripotency.

Transmission of the *Hprt*^Δex7,8^ allele through the germ line and establishment of HPRT-deficient transgenic rats allowed us to examine for the first time, how loss of HPRT activity affected biochemistry in the rat brain. Consistent with the role of HPRT in recycling hypoxanthine and guanine, our analysis revealed changes in levels of compounds associated with purine metabolism. We also detected increases in the amounts of non-purine nucleotides, such as UMP and CMP, in line with elevated levels of pyrimidines previously identified in HPRT-deficient brain tissue and neuronal cell cultures^33^,^34^,^35^. It is noteworthy that the level of orotidine, a derivative of the UMP precursor, orotic acid, was also higher in the HPRT-deficient rat brains. Elevated levels of orotidine have been reported in blood cells of LND patients, and in response to treatment with the drug allopurinol, a hypoxanthine analogue that blocks the breakdown of xanthine to urea^36^,^37^. Although this implicates hypoxanthine as a regulator of pyrimidine metabolism, there was no evidence for higher levels of hypoxanthine in brains of HPRT KO rats. Nevertheless, levels of the second messenger cAMP, which in other experimental systems are affected by hypoxanthine^38^,^39^,^40^, were also increased in HPRT KO rat brains. This increase contrasts with the reduction in cAMP-dependent signalling reported in HPRT-deficient cell lines^41^,^42^, but might reflect the challenges associated with aligning *in vitro* responses with the physiological state *in vivo*. For example, glucose concentration affects the inhibitory effect of hypoxanthine on preimplantation embryonic development^39^,^43^. The changes in core metabolites we report here, such as increases in level of glucose-6 phosphate and citrate, and decreases in lipids and fatty acids, may result from an increased metabolic demand *in vivo* due to compensatory *de novo* nucleotide synthesis in the brain. Perhaps, these direct and indirect consequences of HPRT loss, converge to perturb neural function in the HPRT-deficient brain.

Reduced levels of dopamine and serotonin in the brains of *Hprt*^Δex7,8^ rats confirmed that loss of HPRT activity disturbed normal neuronal function in the rat. The depletion of dopamine in HPRT KO rat brains approached 60% of wild-type levels and given that these brain samples had contributions from overlying cortex, the hippocampus and the striatum, the losses in individual areas might be significantly greater. In human LND patients the reduction in dopamine was 70–90% of normal levels and in HPRT-deficient mice losses ranged between 20–60% depending on the strain, which might impact on modelling neurological deficits associated with LND^4^,^8^,^25^,^44^. Interestingly, the reduced level of serotonin in HPRT KO rat brains was consistent with results from one of the first HPRT KO studies in mice, and points to a broader deregulation of neurotransmitters associated with HPRT loss^25^. Indeed, although a similar deficit was not evident in the post-mortem brains of LND patients^4^, temporary alleviation of self-injurious behaviour in response to treatments that increase serotonin levels, and reduced levels of serotonin receptors in LND patients point to an association between serotonin and HPRT function^45^,^46^. Taken together these results indicate that loss of HPRT activity has wider effects on neurotransmitters than just dopamine, possibly a consequence of the widespread metabolic changes in the HPRT-deficient brain.

In conclusion, although HPRT KO male rats appear generally healthy and fertile, the biochemical analysis of the HPRT-deficient brains pointed to potentially widespread perturbation of general metabolic processes beyond disruption of purine metabolism. Moreover the reduction of dopamine and serotonin within HPRT KO brains mirrors aspects of studies performed on LND patients and HPRT KO mice^4^,^8^,^25^,^44^. The HPRT knock-out rat therefore serves as a new experimental platform to investigate how loss of HPRT activity affects neural function during embryonic development and in adult animals. We suggest that elaboration of this model through the introduction of secondary genetic modifications in associated regulatory pathways should provide further opportunities to dissect mechanisms underlying Lesch-Nyhan Disease.

## Methods

### Ethics statement

Animal work conformed to guidelines for animal husbandry according to the UK Home Office and approval by the Roslin Institute Animal Ethics Committee. Animals were naturally mated and sacrificed under schedule 1, procedures that do not require specific Home Office approval.

### Derivation and culture of rat ES cells

ES cell derivation and culture were as previously described with minor modifications^21^. Cells were cultured on gamma-irradiated (5 Gy) DIA-M or DR4 mouse fibroblasts in: 1i medium; N2B27 + 1 μM PD0325901 (Axon Medchem) or 2i + LIF medium; N2B27 + 1 μM PD0325901 + 3 μM CHIR99021: (Axon Medchem) + 10^3^ units/ml mouse Leukaemia Inhibitory Factor (Millipore).

### Targeting vector construction

A 6.9 kb fragment was amplified from Fischer F344 Rat genomic DNA using oligonucleotides flanking Exons 7, 8 and 9 (HPRTintr6for2 - CCTCCCCAATGCCTACAATG and 3′FLKrev3 – CCTTTCCCTGTCCTACACAC). The PCR was performed using Pfu UltraII Fusion HS DNA Polymerase (Stratagene) under the following conditions; 95 °C for 2 mins, followed by 30 cycles of 94 °C for 30 secs, 60 °C for 30 secs and 68 °C for 10 mins, with a final extension at 68 °C for 10 mins. PCR products were cloned into *Eco*RV digested pBluescript and sequence integrity was confirmed by comparing sequences of four individual clones from separate PCR reactions with the sequence for Brown Norway rat (Ensembl, ENSRNOG00000031367). A 5.4 kb *Bst*BI/*Sac*I fragment was subcloned from the F344 HPRT PCR clone. The 650 bp *Nde*I/*Eco*RV fragment containing exons 7 and 8 was removed and replaced with a selection cassette consisting of PGK*neo* flanked by *frt* recombination sites.

### Electroporation

Approximately 1 × 10^7^ rat ES cells in 0.8 ml phosphate buffered saline (PBS) containing 75 μg *Ahd*I linearised HPRT targeting vector were electroporated using the Bio-Rad Genepulser apparatus (0.8 kV, 3 μF). Electroporated cells were plated into 10 cm^2^ wells containing 2i + LIF (DAK31) or 1i (DA27) medium. The aminoglycoside G418 (150 μg/ml) was added 48 h after electroporation and the number of G418-resistant colonies counted 7 or 8 days later. 6-TG (5 μM) was applied at either day 9 or following picking and replica-plating of individual G418-resistant clones.

### Karyotype analysis

Cells were cultured for two hours in 0.1 mg/ml colcemid (Life Technologies), swollen in 0.56% (w/v) KCl hypotonic solution and fixed on ice in 3:1, methanol:acetic acid. Metaphase spreads were prepared on glass slides and stained with Giemsa (Life Technologies).

### Chimaera generation

Rat blastocysts at E4.5 days post-coitum were cultured for 2–3 hours in M16 embryo culture medium (Sigma) to ensure cavitation, prior to injection. Rat ESC were disaggregated in TVP trypsin solution^22^, pelleted in 1i/2i N2B27 culture medium and pre-plated on gelatin-coated tissue culture plastic in 1i/2i for 45–60 mins. Non-attached cells were pelleted and resuspended in N2B27 containing 20 mM HEPES buffer and kept on ice prior to injection. Blastocysts were transferred to M2 medium (Sigma) before injection with 10–12 cells, and then transferred into the uteri of pseudopregnant Sprague Dawley rats.

### Genomic PCR screening

100 ng of genomic DNA was amplified using oligonucleotides designed to identify the wildtype allele (Ex7for - GTGTTGGATACAGGCCAGAC and Ex8rev - GTGCTCATTATAGTCAAGGG) and the targeted allele (HPRT2524for – CTTCTCCCTTTCAGTCTTCC and PGKrev – GATGTGGAATGTGTGCGAGG). The PCR was performed using FastStart Taq (Roche) under the following conditions; 95 °C for 2 mins, followed by 33 cycles of 95 °C for 30 secs, 55 °C for 45 secs and 72 °C for 10 mins, with a final extension at 72 °C for 10 mins. Products were separated by 1% TBE agarose gel electrophoresis and visualised with ethidium bromide. The expected sizes of the wildtype and targeted alleles are 252 bp and 286 bp respectively.

### Southern Blot

Eight to ten micrograms of genomic DNA were digested with 200 units of restriction enzyme at 37 °C for 30 hours. The resulting DNA fragments were resolved on a 0.7% TAE agarose gel overnight at 25 V. The DNA fragments were UV-nicked prior to transfer to Hybond N + Nylon membrane (GE Healthcare, RPN203B) as described in the manufacturer’s instructions. Following transfer, the DNA was UV cross-linked on to the membrane. Probes were prepared by PCR amplification of HPRT sequence flanking the 5′ and 3′ homology arms (Intr6F2 – CCTCCCCAATGCCTACAATG and HPRT289R – GAAAAAGGAAGCAAGTGTGG, and HPRT6125F – GTGCTGTTTTCCTCATGGGC and HPRT6373R – GCTACCTTCTGGCTTTGTTAG for 5′ and 3′ probes respectively).

25 ng of probe DNA was radioactively labelled with α–dCTP P^32^ using High Prime (Roche, 11 585 592 001), then hybridised to the membrane overnight at 65 °C in Church solution containing 10 μg/ml sonicated Herring Sperm DNA and 10 μg/ml tRNA. Non-specifically bound probe was removed by washing in 2× SSC/0.1% (w/v) SDS at 65 °C. The membrane was exposed to Kodak Biomax MS film at −80 °C.

### Western Blotting

Western blot analysis was performed using Novex BisTris 12% precast PAGE gels run in MOPS buffer, transferred to Hybond-ECL nitrocellulose membranes, consecutively probed with primary antibodies and fluorescently labelled secondary antibodies, diluted in Li-Cor blocking buffer and scanned using a Li-Cor Odyssey Scanner as recommended by the manufacturers. Antibodies used were: anti-HPRT (Abcam ab10479) and anti- γ-tubulin mab (Sigma-Aldrich, T5326).

### Histology

Free floating immunohistochemistry was performed on brains from wild-type and HPRT-knockout adult male rats. Rats were kept in single cages with food and water ad libitum on 12-h light/dark cycle (lights on 0700 h). The rats were killed between 2 and 5 h after lights on, and their brains were removed and hemi-dissected in the sagittal plane through the midline (Supplementary Fig. S3). Half of the brain was immersion fixed in 4% (w/v) paraformaldehyde in 0.1 M PBS solution (pH 7.4) for 7 days, post-fixed in 15% (w/v) sucrose in 4% (w/v) PFA overnight, cryoprotected in 30% (w/v) sucrose in 0.1 M PBS for a further 24 hours and snap-frozen on dry ice. The other half of the brain was used in the HPLC metabolomics study (see below). The brain was sectioned in the coronal plane at 50 μm on a freezing microtome and then processed for immunohistochemistry using a mouse anti-tyrosine hydroxylase (TH) monoclonal antibody (Millipore). The antibody was diluted to a concentration of 1:1000 in phosphate buffer (PB) with 0.2% (v/v) Triton X-100 and 3% (v/v) normal goat serum. Sections were incubated in TH antibody for 24 h at 4 °C. The antibody-antigen complex was localized and amplified using the ABC method with a Vector elite kit (Vector Inc). TH immunoreactivity was visualized using 0.025% (w/v) diaminobenzidine with 0.03% (v/v) hydrogen peroxide, adapted from^47^. Following washes in PB, sections were mounted onto gelatin-coated slides, air dried, dehydrated through an alcohol series and sealed with using a coverslip with Pertex mounting medium (Leica Microsystems).

### Analysis of Uric Acid in rat serum

Rat blood was harvested immediately from freshly culled and decapitated adult rats. 1 ml of blood was placed in a 1.5 ml-coated clotting tube, mixed gently by inversion and incubated for 45 minutes at room temperature. Serum was harvested by centrifugation for 15 minutes at 1300 × *g*. Serum was carefully removed by pipette and transferred to a fresh tube. Cellular debris was removed by centrifugation for a further 15 minutes at 1300 × *g*. Uric acid analysis was performed using a colorimetric peroxidase-coupled indirect equilibrium uricase method by Pinmoore Animal Laboratory Services Limited, Cheshire, UK.

### Quantitation of selected neurochemicals

Prior to analysis, 16 brains (9 control, 7 knock-out) were sectioned into 6 areas according to the scheme shown in Supplementary Fig. S4. The basic methodology for neurochemical quantitation follows that published previously^48^. Briefly, brain samples were homogenised in 20 mM Ascorbic acid (100 μl per 50 mg tissue) using a handheld homogeniser (Fisher). To 125 μl of homogenate was added 2 μl of a 10 ng/μl solution of caffeic acid, which acted as an internal standard to control for variations in extraction, and 500 μl ice cold acetonitrile. The sample was vortexed and the precipitate removed by centrifugation (13,000 × g for 15 mins at 4 °C). The supernatant was evaporated under vacuum and the residue resuspended in 25 μl of labelling buffer (100 mM sodium tetraborate). 25 μl of labelling reagent (2% (v/v) benzoyl chloride in acetonitrile) was added and the reaction mixed at room temperature. Insoluble material was removed by centrifugation (13,000 × g for 10 minutes at 4 °C) and the supernatant analysed by HPLC-MS.

Neurochemicals were separated by use of an Ascentis Express HPLC column (C_18_, 2.1 mm id × 15 cm length, 2.7 μm bead size, Supelco) attached to a Dionex Ultimate HPLC system. The eluent was pumped through the column at a flow rate of 200 μl/min (solvent A was 10 mM ammonium formate & 0.15% formic acid, solvent B was acetonitrile) and the separation was developed by a multipart gradient as follows: 0 mins – 20% B; 0.5 mins – 20% B; 2 mins – 45% B; 7.5 mins – 64% B; 8 mins – 100% B. This gradient was followed by a 3.5 minute hold at 100% B, followed by a reduction to 20% B and a 1 minute hold to re-equilibrate the column. The eluent was passed directly to the electrospray source of an Amazon ETD ion trap mass spectrometer (Bruker) operated in +ve ion, multiple reaction monitoring mode. MS conditions for each analyte are provided (Supplementary Table S7). At least 6-point standard curves of pre-labelled analytes in solvent A were built and samples were run in at least duplicate, in 7 batches. Data processing was performed using the Bruker Compass plugin, QuantAnalysis. Any effect of matrix on measurement of neurochemicals in the brain samples was corrected for by use of measured concentration of the internal standard, caffeic acid. Finally repeat measurements were averaged, and significant differences in the concentration of different analytes between KO and WT samples were revealed by student t-tests.

### Metabolomic workflow, data acquisition and analysis

Dissected brain samples were flash frozen in liquid N_2_ and ground to a powder using a chilled pestle and mortar. Approximately 5 μl volume of ground sample was solubilised in 200 μl of chloroform/methanol/water solution (1:3:1) at 4 °C by vortexing. The lysate was mixed at 4 °C for 1 hr and then centrifuged for 3 minutes at 13,000 g at 4 °C. Approximately 180 μl of the cleared supernatant was stored at −80 °C prior to analysis by MS.

### LC-MS

Samples (10 μl) were analysed by hydrophilic interaction liquid chromatography (HILIC)-mass spectrometry (LC-MS) (UltiMate 3000 RSLC (Thermo Fisher, San Jose, California, USA) using a 150 × 4.6 mm ZIC-pHILIC column (Merck SeQuant, Umea, Sweden) running at 300 ul/min and an Exactive (Thermo Scientific) for MS detection. Buffers consisted of A: 20 mM ammonium carbonate (Sigma Aldrich) in H^2^O (Fisher Scientific) and B: acetonitrile (Fisher Scientific). The gradient ran from 20% A: 80% B to 80% A: 20% B in 15 min, followed by a wash at 95% A: 5% B for 3 min, and equilibration at 20% A: 80% B for 5 min. The Exactive was used to detect ions using a HESI-II interface with a source temperature of 150 °C and capillary temperature of 270 °C. Sheath gas flow rate was 40, Aux gas flow rate was 5 and sweep gas was 1. Each sample was injected simultaneous detection in positive ion and negative ion using polarity switching mode, the spray voltage was 4.5 kV and 3.5 kV respectively. Full scan MS detection was acquired for the range 70–1400 m/z at 50000 (at 400 m/z) resolution mode for each polarity. Raw mass spectrometry data was processed using our standard pipeline, consisting of XCMS (for peak picking), MzMatch (for filtering and grouping) and IDEOM (for further filtering, post-processing and identification). Core metabolite identifications were validated against a panel of unambiguous standards using accurate mass and retention time. Additional putative identifications were assigned by accurate mass along with a retention time prediction algorithm.

### LC-MS/MS

Samples (10 μl) were separated by a SeaQuant ZIC-pHILIC 4.6 × 150 mm column (Merck) at a flow rate of 0.300 ml/min mobile phase. A gradient from 80% acetonitrile/20% 10 mM ammonium carbonate to 5% Acetonitrile/95% ammonium carbonate over 15 min was used to elute compounds. A 2 min hold at 5% acetonitrile was used to wash the column. The solvents were returned to initial conditions and held for 7 min for column re-equilibration. A QExactive (Thermo Scientific) was used to detect ions using a HESI-II interface. A source temperature of 150 °C and capillary temperature of 320 °C was used. Sheath gas, auxiliary gas and sweep gas flow rates of 40, 5 and 1 L/min were used. Polarity switching mode was used for detection. The spray voltage was 3.8 kV in either polarity. Full scan MS spectra were acquired for the range 70–1050 m/z with a resolution of 70000 (at 400 m/z). A maximum of 5 ions selected from the precursor ion list were selected for MS/MS and were isolated using a 2 m/z window and activated with HCD at 60.0 NCE. Fragment ion spectra were acquired using a resolution of 35000 (at m/z 400). Fragment ion spectra were searched against online public databases, Massbank (http://www.massbank.jp/), mzCloud (https://mzcloud.org/) and Metlin (http://metlin.scripps.edu) and in silico fragmentation using MetFrag (http://msbi.ipb-halle.de/MetFrag/) searched against Kegg database. When no compounds were found from Kegg then PubChem and Chemspider was used as an alternative. All databases were searched using 20 ppm mass window for the fragmentation data.

### Statistical Analysis

Metabolites identified in the LC-MS analysis were filtered to identify those that differed statistically (t-test p ≤ 0.05) in level between wild-type and mutant animals in the forebrain (region B) and central region (region A) (Supplementary Tables S2, S3). The analyses were performed using two-tailed t-tests and normality assumptions for this test were satisfied. The selected metabolites that had elements within their MS/MS spectra that were consistent with molecular assignments made in the initial LC-MS analysis were analysed using ANOVA models to test the type (wild-type or HPRT KO) by area (regions A or B) interaction. This determined whether there was a significant difference (p ≤ 0.05) in the type effect between the brain areas. Where the type interaction was non-significant then the average of the region A and B brain areas was compared between the wild-type and mutant groups using a two-tailed t-test (Supplementary Table S8). Comparing the average level of validated standard compounds in wild-type and HPRT KO brains indicated there were increased levels of UMP, CMP, cAMP and glucose-6-phosphate in the brains of HPRT KO animals.

## Additional Information

**How to cite this article**: Meek, S. *et al*. Reduced levels of dopamine and altered metabolism in brains of HPRT knock-out rats: a new rodent model of Lesch-Nyhan Disease. *Sci. Rep.* **6**, 25592; doi: 10.1038/srep25592 (2016).

## Supplementary Material

Supplementary Information

Supplementary Tables

## Figures and Tables

**Table 1:** Altered levels of 20 metabolites in brains of HPRT knock-out rats.

**Kegg ID**	**Compound**	**Pathway**	Validation level*	**Region A KO:WT ratio**	**Region A t-test**	**Region B KO:WT ratio**	**Region B t-test**
C02718	N-Formiminoglycine	nucleotide metabolism	3	n/a	0.003	30.545	0.001
–	Orotidine	nucleotide metabolism	1	2.417	0.012	2.903	0.028
C03366	5-Phosphonooxy-L-lysine	amino acid metabolism	3	n/a	0.010	n/a	0.006
C01035	4-Guanidinobutanoate	amino acid metabolism	2	1.431	0.020	1.542	0.016
C00158	Citrate	carbohydrate metabolism	1	1.792	0.026	2.210	0.036
C00416	LPA(0:0/18:1(9Z))	lipid metabolism	2	0.681	0.048	0.660	0.008
C06424	Tetradecanoic acid	lipid metabolism	3	0.654	0.016	0.674	0.015
C08362	(9Z)-Hexadecenoic acid	lipid metabolism	3	0.533	0.008	0.660	0.017
C00712	[FA (18:1)] 9Z-octadecenoic acid	lipids: fatty Acyls	3	0.489	0.019	0.645	0.014
C06429	Docosahexaenoicacid	lipids: fatty Acyls	2	0.452	0.040	0.567	0.005
C16522	Icosatrienoic acid	lipids: fatty Acyls	2	0.376	0.010	0.515	0.010
C16527	[FA (22:4)] 7Z,10Z,13Z,16Z-docosatetraenoic acid	lipids: fatty Acyls	2	0.355	0.023	0.551	0.005
C11896	Docosapentaenoic acid (Taxa-4(20),11(12)-dien-5alpha-yl acetate)	lipids: fatty Acyls	2	0.333	0.021	0.437	0.001
–	[GP (20:4)] 1-(5Z,8Z,11Z,14Z-eicosatetraenoyl)-sn-glycero-3-phosphate	lipids: glycerophospholipids	3	0.509	0.012	0.543	< 0.001
–	LysoPE(0:0/22:4(7Z,10Z,13Z,16Z))	lipids: glycerophospholipids	3	0.415	0.045	0.643	0.037
–	LysoPE(0:0/22:6(4Z,7Z,10Z,13Z,16Z,19Z))	lipids: glycerophospholipids	3	0.345	0.006	0.482	0.005
–	[PE (20:4)] 1-(5Z,8Z,11Z,14Z-eicosatetraenoyl)-sn-glycero-3-phosphoethanolamine	lipids: glycerophospholipids	3	0.338	0.008	0.439	0.001
C16621	1-Methyl-4-nitroimidazole	n/a	3	n/a	0.000	n/a	0.008
–	DL-2-Aminooctanoicacid	n/a	2	3.038	0.033	3.549	0.034
C01546	2-Furoate	n/a	2	1.802	0.013	2.381	0.030

The changes in levels of 20 metabolites that differed in both regions A (Cortex, hippocampus, striatum and hypothalamus) and region B (olfactory bulb and pre-frontal cortex) of HPRT-deficient rat brains. The MS/MS spectra of these compounds had elements that were consistent with molecular assignments made in the LC-MS analysis. Validation confidence levels are 1. Match to a known standard; 2. MS/MS data matches to a public library with score of >0); 3. MS/MS data matches by *In Silico* (MetFrag) approach with score > 0).
