# Supplementary material for: Reduced levels of dopamine and altered metabolism in brains of HPRT knock-out rats: a new rodent model of Lesch-Nyhan Disease
Source: Sci Rep. 2016 May 17;6:25592. doi: 10.1038/srep25592 (PMC4869022; doi:10.1038/srep25592)
Supplement: Supplementary Information [file srep25592-s1.pdf]

Reduced levels of dopamine and altered metabolism in brains of HPRT knock-out rats: a new rodent model of Lesch-Nyhan Disease.

Stephen Meek[1], Alison J. Thomson[1], Linda Sutherland[1], Matthew G.F. Sharp[2], Julie Thomson[2], Valerie Bishop[1], Simone L. Meddle[1], Yoann Gloaguen[3], Stefan Weidt[3], Karamjit Singh-Dolt[1], Mia Buehr[1], Helen K. Brown[1], Andrew C. Gill[1,4], and Tom Burdon[1,4]

[1] The Roslin Institute and R(D)VS, University of Edinburgh, Easter Bush, Midlothian, EH25 9RG

[2] Central Bioresearch Services, University of Edinburgh, Chancellor's Building, 49 Little France Crescent, Edinburgh EH16 4SB

[3] Glasgow Polyomics, College of Medical, Veterinary and Life Sciences, University of Glasgow, Wolfson Wohl Cancer Research Centre, Garscube Campus, Bearsden, G61 1QH

[4] Corresponding authors:

Tom Burdon, Tel: +44 (0)131 6519169: tom.burdon@roslin.ed.ac.uk.

Andrew Gill, Tel: +44 (0)131 6519121: andrew.gill@roslin.ed.ac.uk

AJT current address: New World Laboratories, 500 Boulevard Cartier Ouest, Laval, Quebec, H7V 5B7, Canada,

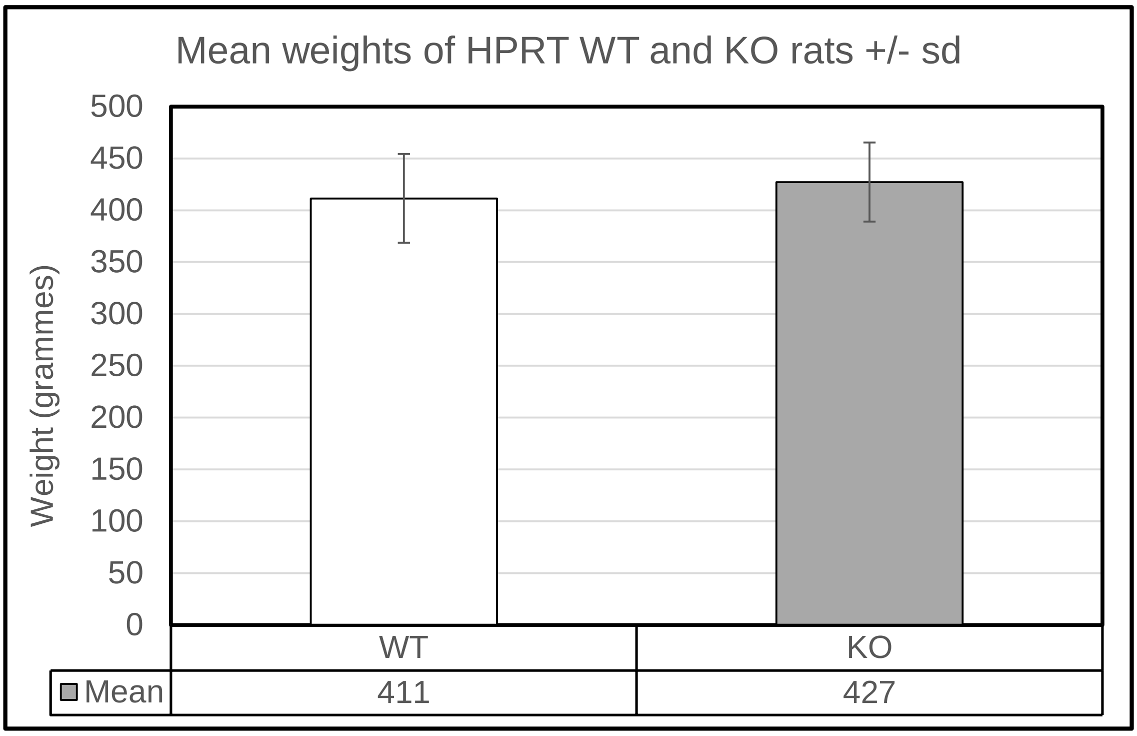

Meek et al., Supplementary Figure S1. Average weight of male HPRT Wild-type and knock-out rats. Graph of mean weights of 9 HPRT WT and 7 KO male rats +/- SD. The ages of rats ranged from 9-15 months.

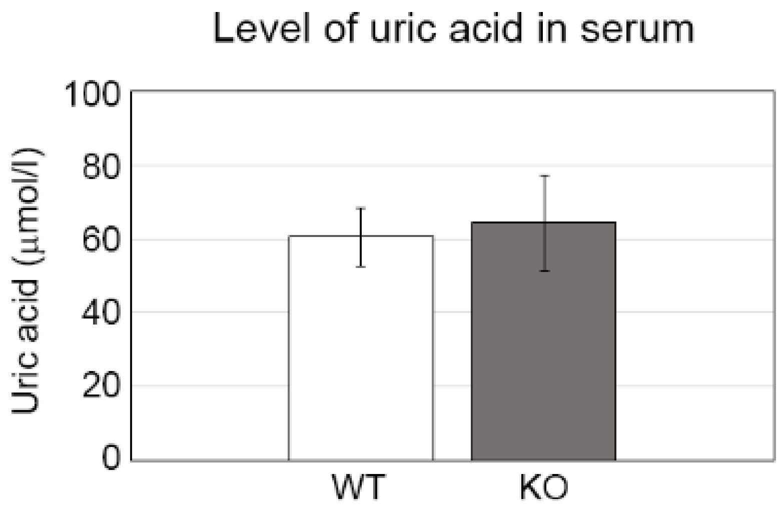

Meek et al., Supplementary Figure S2. Uric acid levels in HPRT-deficient rats. Analysis of uric acid levels in blood serum harvested from four HPRT wildtype (WT) and five HPRT-deficient male rats (KO). The average measurement for each group are presented +/- SD. The ages of rats ranged from 9-12 months.

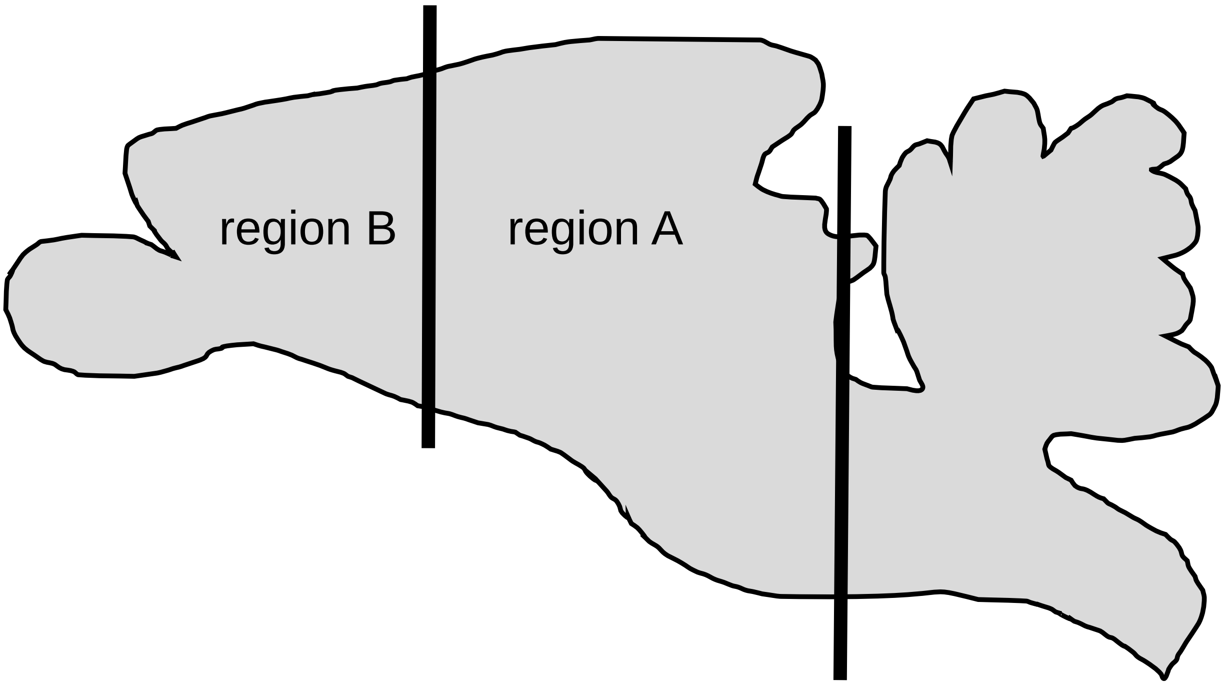

Meek et al., Supplementary Figure S3. Sagittal diagram of rat brain dissection used in metabolomics analysis. Region A contains Cortex, Hippocampus, Striatum and Hypothalamus. Region B contains Olfactory bulb and Pre-frontal cortex. Black bars identify where the brain was sectioned.

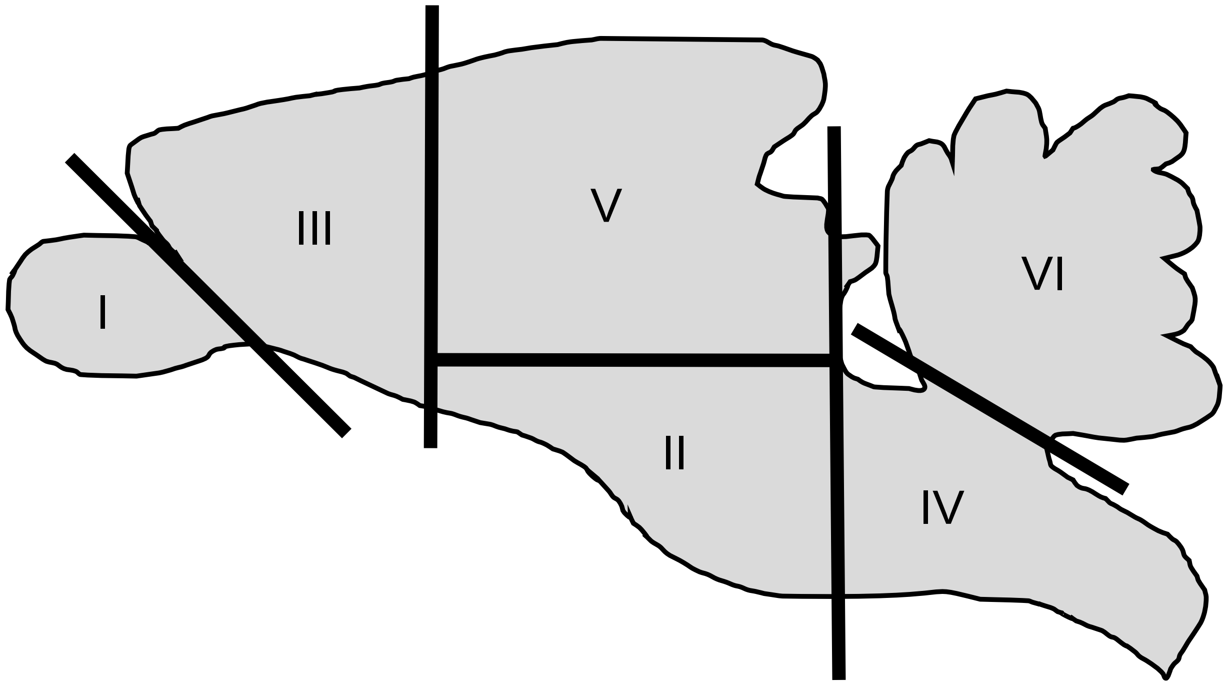

Meek et al., Supplementary Figure S4. Sagittal diagram of rat brain dissection used in neurotransmitter HPLC-MS analysis. Regions I is olfactory bulb, II: Hypothalamus; III: Pre-frontal cortex; IV: Midbrain; V: Hippocampus, Cortex and Striatum; VI: Cerebellum. Black bars identify where the brain was sectioned.

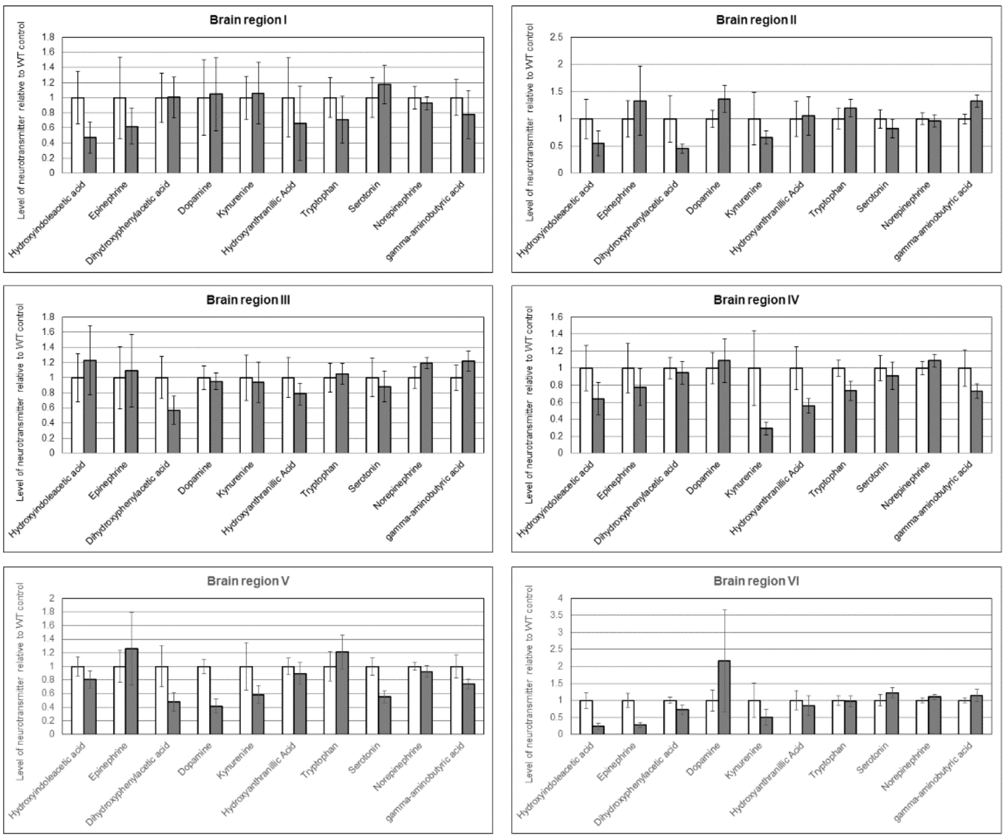

Meek et al., Supplementary Figure S5. HPLC-MS analysis of neurotransmitters in brains of HPRT wild-type and knock-out rats. Brain regions correspond to: I: Olfactory bulb; II: hypothalamus; III: Pre-frontal Cortex; IV: Midbrain; V: Hippocampus, cortex and striatum; VI: Cerebellum. Graphs present mean levels normalised relative to mean levels in wild-type rats +/- SEM. White bars represent wild-type values and grey bars are the knock-outs.

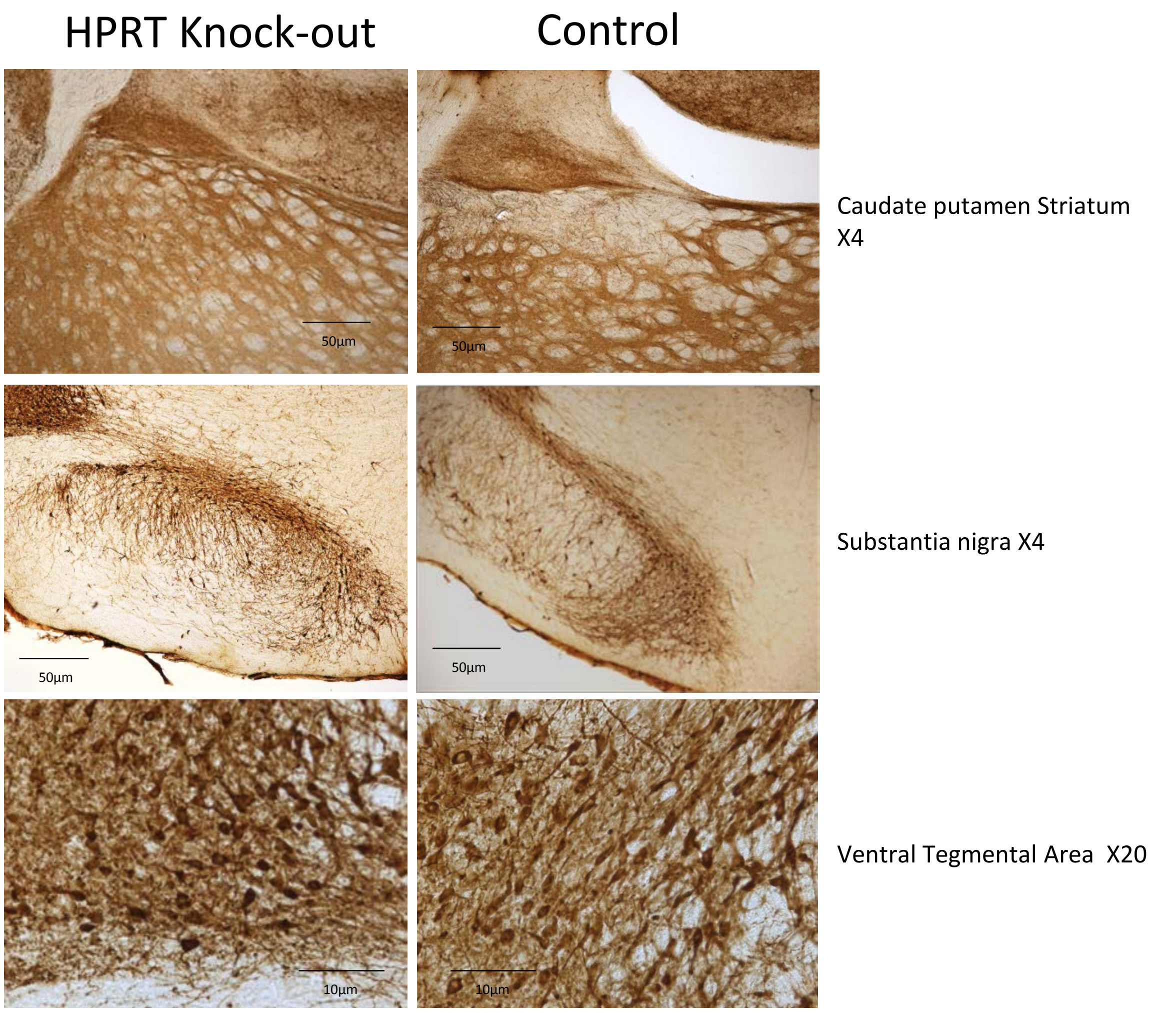

Meek et al., Supplementary Figure S6. Tyrosine Hydroxylase (TH) immunohistochemistry of coronal brain sections of HPRT knock-out and wildtype control rats. The localisation of TH was performed using the ABC method and developed using diaminobenzidine and hydrogen peroxide to form a brown precipitate. The TH expressing neurons in the base of the forebrain (caudate putamen striatum) and midbrain (substantia nigra and ventral tegmental area) are shown.
